# Supplementary material for: Homocystamide Conjugates of Human Serum Albumin as a Platform to Prepare Bimodal Multidrug Delivery Systems for Boron Neutron Capture Therapy
Source: Molecules. 2021 Oct 29;26(21):6537. doi: 10.3390/molecules26216537 (PMC8586956; doi:10.3390/molecules26216537)
Supplement: Supplementary file 1 [file molecules-26-06537-s001.zip › molecules-1423439-supplementary.pdf]

Electronic Supplementary Information

Homocystamide conjugates of human serum albumin as a platform to prepare bimodal multidrug delivery systems for boron-neutron capture therapy

Tatyana V. Popova,[1,2] Maya A. Dymova,[1] Ludmila S. Koroleva, [1] Olga D. Zakharova,[1] Vladimir A. Lisitskiy,[1] Valeria I. Raskolupova,[1,2] Tatyana V. Sycheva,[3] Sergei Yu. Taskaev,[2,3] Vladimir N. Silnikov,[1] Tatyana S. Godovikova[1,2,#]

[1] Institute of Chemical Biology and Fundamental Medicine, SB RAS, 630090 Novosibirsk, Russia
[2] Novosibirsk State University, 630090 Novosibirsk, Russia
[3] Budker Institute of Nuclear Physics, SB RAS, 630090 Novosibirsk, Russia
* Correspondence: Dr. Tatyana S. Godovikova, t_godovikova@mail.ru; Tel.: 8-383-3635183

Table of contents

1	General remarks	S2
2	Synthetic procedures	S3
2.1	Synthesis of maleimide-conjugating closo-dodecaborate tetramethylammonium form ($B_{12}H_{11}$-mal)	S3
2.2	Synthesis of 2,2,2-trifluoro-*N*-(2-oxotetrahydrothiophen-3-yl)acetamide (HTLTFAc)	S4
3	Bioconjugation	S4
3.1	Synthesis of HSA-Cy5, HSA-Cy5-HcyTFAc and HSA-Cy5-HcyAc conjugates	S4
4	Characterization of homocystamide conjugates of human serum albumin with MALDI-ToF mass spectrometry	S5
4.1	HSA-Cy5-HcyTFAc conjugate	S5
4.2	HSA-Cy5-HcyAc conjugate	S6
5	Gel retardation assay of boron-albumin conjugates	S6
6	Circular dichroism data	S7
7	NMR data	S8
	REFERENCES	S11
	Table S1	S5
	Table S2	S7
	Table S3	S7
	Scheme S1	S3
	Scheme S2	S4
	Figure S1	S5
	Figure S2	S6
	Figure S3	S7
	Figure S4	S7
	Figure S5	S9
	Figure S6	S10
	Figure S7	S11
	Figure S8	S11

1. General remarks

Human serum albumin (HSA) was obtained from Sigma–Aldrich Chem. Co. (St. Louis, MO, USA). The product number of HSA used was A3782. The SH contents of albumin and albumin products were determined using the Ellman's method as describes in the literature at pH 8 and employed DTNB (5,5′-dithio-bis(2-nitrobenzoic acid) spectrophotometrically at 412 nm (ε = 1.36 × 10^4 $M^{-1}cm^{-1}$) [1]. The concentrations of albumin solutions were determined by absorption at 292 nm, pH 13, using the molar extinction coefficient ε = 4.44 × 10^4 $M^{-1}cm^{-1}$ [2].

Reagents and materials were purchased from Sigma-Aldrich (St. Louis, USA), unless otherwise indicated. Milli-Q water with conductivity greater than 18MΩ/cm was used in all experiments. Phosphate buffered saline (PBS) (0.01 M, pH 7.3–7.5, Biolot).

Boronophenylalanine was purchased from Katchem Ltd (Czech Republic). The enrichment of ^{10}B was ≥99.6%. Boronophenylalanine of 500 mg was mixed with 1100 mg of fructose, 15 ml of H_2O (Milli-Q Water, USA) and 2.7 mL of 1 M NaOH, neutralized with hydrochloride to pH 7.2. Final concentration of fructose-boronophenylalanine was approximately 1100 µg of ^{10}B/ml.

Electronic absorption spectra were acquired on a UV-1800 spectrometer (Shimadzu, Japan).

NMR spectra. ^{19}F NMR spectra were recorded on AV-300 NMR spectrometer (Bruker, Germany) at 282.7 MHz. ^{1}H and ^{13}C NMR spectra were recorded on an AV-400 spectrometer (Bruker, Rheinstetten, Germany) at 400.13 and 100.61 MHz frequency, respectively. The spectra were detected at 25°C in 5 mm NMR sample tubes. All ^{1}H chemical shifts were calculated relative to the residual ^{1}H NMR signal of the deuterated NMR solvents (D_2O, δ 4.80 ppm; DMSO-d6, δ 2.5 ppm; $CDCl_3$, δ 7.3 ppm; acetone-D6, δ = 2.00). C_6F_6 (δ 0.00 ppm) was used as an external reference for chemical shifts in ^{19}F NMR spectra. The ^{13}C chemical shifts are reported relative to the solvent (CD_3OD, δ = 49.3 ppm). Chemical shifts (δ) are reported in parts per million (ppm) and coupling constants (J) in Hertz (Hz). The multiplicity of each signal is indicated as s-singlet, d-doublet, t-triplet, m-multiplet (i.e. complex peak obtained due to overlap) or a combination of these. Broad peaks are indicated by the addition of br.

Inductively coupled plasma atomic emission spectroscopy was performed using ICPE-9820 (Shimadzu, Japan). A conjugate sample in PBS (40 µL, 0.4 mM) was diluted to 4 mL with bidistillated water and used for measurement.

Mass spectra of the low molecular weight compounds were recorded on Ion trap XTC ultra (Agilent Technologies, USA) mass spectrometer with an electrospray ionization (ESI) interface.

Mass spectra of proteins and peptides were recorded on Bruker Autoflex Speed (Bruker Daltonics, Germany) MALDI-ToF mass spectrometer in a positive linear mode. A smartbeam-II laser was used. 2,5-Dihydroxyacetophenone (2,5-DHAP) was used as a matrix. Protein samples were desalted by ZipTip C4 pipette tips. Peptide samples were desalted by ZipTip C18 pipette tips. A 2 µL of the sample solution was mixed with 2 µL of a 2% TFA (trifluoroacetic acid). To the latter solution 2 µL of the matrix (2,5-DHAP) was added. The mixture was pipetted up and down until the crystallization starts. Mass spectra were obtained by averaging 3,000 laser shots. External calibration was provided by $[M + H]^+$ HSA at m/z 66.5 kDa.

2. Synthetic procedures

All reactions were carried out at atmospheric pressure. Room temperature is defined as between 19–21°C. The term *in vacuo* refers to solvent removal using Büchi rotary evaporation between 15–60°C, at approximately 10 mm Hg.

2.1. Synthesis of maleimide-conjugating closo-dodecaborate tetramethylammonium form ($B_{12}H_{11}$-mal, Scheme S1)

Compound $B_{12}H_{11}$-mal was synthesized according to the literature procedure [3]. The synthesis of $B_{12}H_{11}$-mal is shown in Scheme S1.

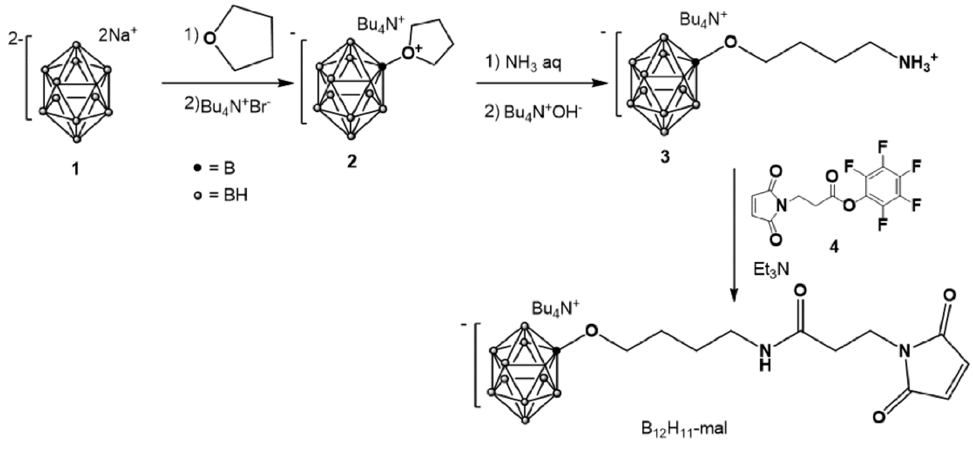

Scheme S1

The key to the functionalization at B-H of *closo*-dodecaborate is the formation of a cyclic oxonium complex (**2**) [4], followed by the nucleophilic ring-opening reaction reported *Sivaev et al.* [5]. Briefly, *closo*-dodecaborate (**1**) was reacted with tetrahydrofuran in the presence of BF_3 to form *closo*-dodecaborate cyclic oxonium complex (**2**), which underwent the ring-opening reaction with aqueous ammonium in the presence of tetrabutylamine to give corresponding compound (**3**) in 89% yield. Resulting amine (**3**) was reacted with 2-maleimidoethanic acid pentafluorophenyl ester to give maleimide-functionalized *close*-dodecaborate ($B_{12}H_{11}$-mal) in 31.8% yield.

Tetrahydrofurane-*closo*-dodecaborate complex (**2**). The yield 84%. ^{1}H NMR ($CDCl_3$, ppm): 4.52 (4H, dt, –O(CH_2CH_2)$_2$), 3.21 (8H, m, Bu_4N^+), 2.15 (4H, dt, –O(CH_2CH_2)$_2$), 1.62 (8H, m, Bu_4N^+), 1.44 (8H, q, Bu_4N^+), 0.98 (12H, t, Bu_4N^+).

Bis-tetrabutylammonium-(4-aminobuthoxy)-undecahydro-*closo*-dodecaborate (**3**). The yield 89 %. ^{1}H NMR (acetone-d6, ppm): 3.60 (2H, t, -CH_2OB_{12}); 3.42(16H, m, $N^+CH_2CH_2CH_2CH_3$); 3.08 (2H, m, -CH_2NH_2); 1.79 (20 H, m, $OCH_2CH_2CH_2CH_2N$ and $N^+CH_2CH_2CH_2CH_3$); 1.43 (16H, m, $N^+CH_2CH_2CH_2CH_3$); 0.97 (24H, t, $N^+CH_2CH_2CH_2CH_3$).

Maleimide-conjugating *closo*-dodecaborate tetramethylammonium form ($B_{12}H_{11}$-mal). The yield: 35.3 mg (31.8 %), R_f 0.67 (CH_2Cl_2:MeOH, 4:1); ^{1}H NMR ($CDCl_3$, δ, ppm): 6.62 (2H, s, CH); 3.75 (2H, t, J = 7.1 Hz, CH_2); 3.54 (2H, m,CH_2); 3.21 (8H, m, $N^+CH_2CH_2CH_2CH_3$); 2.57 (2H, m, CH_2); 1.58 (12 H, m, $OCH_2CH_2CH_2CH_2N$ and $N^+CH_2CH_2CH_2CH_3$); 1.39 (10H, m, $OCH_2CH_2CH_2CH_2N$ and $N^+CH_2CH_2CH_2CH_3$); 0.93 (12H, t, J 7.3, $N^+CH_2CH_2CH_2CH_3$). ^{13}C NMR ($CDCl_3$): 170.52, 170.23, 133.99, 67.99, 58.52, 37.95, 34.88, 34.03, 29.39, 26.74, 23.87, 19.45, 13.55. ESI-MS *m*/*z* 622.30 [M+Bu_4N] $C_{27}H_{62}B_{12}N_3O_4$. calcd. for *m*/*z* 622.53 [M+Bu_4N].

2.2. Synthesis of 2,2,2-trifluoro-N-(2-oxotetrahydrothiophen-3-yl)acetamide (HTLTFAc, Scheme S2)

Compound 2,2,2-trifluoro-*N*-(2-oxotetrahydrothiophen-3-yl)acetamide (HTLTFAc) was prepared according to the literature reported procedure [6]. The yield: 52%. R_f = 0.75. ^{1}H NMR (acetone–D6, δ, ppm, *J*, Hz): 4.8 (q, 1H, 2-H, $J_{2,3\alpha}$ = 7.0); 3.53 (m, 1H, 4α-H); 3.39 (m, 1H, 4β-H); 2.68 (m, 1H, 3β-H); 2.36 (m, 1H, 3α-H). ^{13}C NMR (acetone-D6, δ ppm, *J*, Hz): 203.6 (s, 1-C); 159.8 (q, *J* = 33 Hz, 5-C); 118 (m, 6-C); 69.6 (c, 2-C); 43.2 (c, 4-C); 30.3 (c, 3-C). ^{19}F NMR (acetone–D6, δ, ppm): 88.4 (s, CF_3).

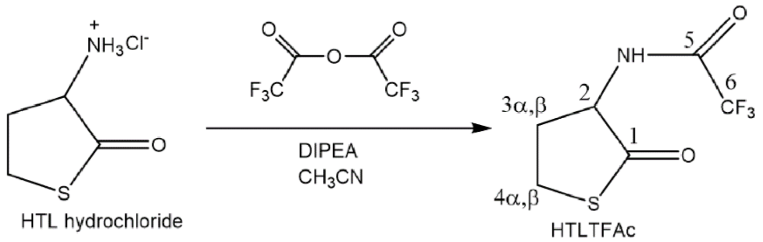

Scheme S2

3. Bioconjugation

Conjugation experiments were carried out in standard polypropylene Eppendorf® safe-lock tubes (1.5 mL) at atmospheric pressure with mixing at 37 °C unless otherwise stated. All buffer solutions were prepared with doubly deionized water. Conjugation buffer was phosphate-buffered saline (PBS) (12 mM phosphates, 140 mM NaCl at pH 7.4).

Low molecular weight materials (MW< 3 kDa) were removed from solutions of polymer conjugates by centrifugal filtration using Centricon concentrators with a MWCO of 3-kDa (Amicon Centriprep YM30, Millipore, Bedford, MA).

3.1. Synthesis of HSA-Cy5, HSA-Cy5-HcyTFAc and HSA-Cy5-HcyAc conjugates (Figure 1)

The synthesis of HSA-Cy5, HSA-Cy5-HcyTFAc and HSA-Cy5-HcyAc conjugates was adapted from was adapted from *Chubarov et al.* [7].

Briefly, HSA (133 mg, 2 μmol) was dissolved in PBS buffer (2 mL, 1 mM) and mixed with threefold excess of sulfo-Cy5 maleimide derivative (1 μmol, 0.8 mg) dissolved in 0.1 mL DMSO. The reaction was carried out overnight while protected from light. Ellman's test showed that no free sulfhydryl groups were left. Subsequently, low molecular weight materials were removed from solutions of protein conjugates by ultrafiltration at 9000 rpm twenty-four times using a Millipore ultrafiltration tube (Amicon Centriprep YM30, Millipore, Bedford, MA).

HSA-Cy5 was used in the next reaction step. The solution of HTLTAc (5.46 μmol, 1.4 mg) or HTLAc (5.46 μmol, 0.87 mg) in DMSO (0.05 mL) was added to the solution of HSA-Cy5 conjugate in PBS buffer (pH 7.4) (1 mL, 56.5 mg, 0.84 μmol). The reaction mixture was incubated under constant gently stirring at 37 °C in the dark for 42 h. The protein conjugates were purified by SEC utilizing a Millipore ultrafiltration tube and stored at 4 °C.

The yield of HSA-Cy5-HcyTFAc derivatives was ~55%. UV-vis (PBS buffer, pH 7.4): λmax 278 nm (ε = (3.88 ± 0.1) × 10^4), λ_{max} 650 nm (ε = (6.96 ± 0.1) × 10^4).

The yield of HSA-Cy5-HcyAc was ~65%. UV-vis (PBS buffer, pH 7.4): λ_{max} 278 nm (ε = (3.88 ± 0.1) × 10^4), λ_{max} 650 nm (ε = (5.05 ± 0.1) × 10^4).

The incorporation of homocysteine residues into albumin was proved by Ellman's assay [1] taking HSA-Cy5 as control. The SH contents of albumin product was determined at pH 8 and employed DTNB spectrophotometrically at 412 nm (ε = (1.36 ± 0.1) × 10^4 $M^{-1}cm^{-1}$) with a UV-1800 spectrometer (Shimadzu, Japan). The amount of additional free sulfhydryl groups per protein molecule (modification degree) for the

proteins was calculated to be 3.0 ± 0.1 and 1.8 ± 0.2 for the HSA-Cy5-HcyTFAc and HSA-Cy5-HcyAc, respectively.

4. Characterization of homocystamide conjugates of human serum albumin with MALDI-ToF mass spectrometry

Changes in molecular mass of albumin conjugates was monitored with MALDI-ToF mass spectrometry. The ToF mass analyzers used in these experiments did not measure the *m/z* values for $[M + H]^+$ ions in MALDI spectra with great accuracy for masses over 60 kDa. MALDI mass spectra for each sample were recorded in four replicates with *m/z* values that differed by 20–100 Da. Additionally, the different post-translationally modified forms caused spectral overlap.

4.1. HSA-Cy5-HcyTFAc conjugate

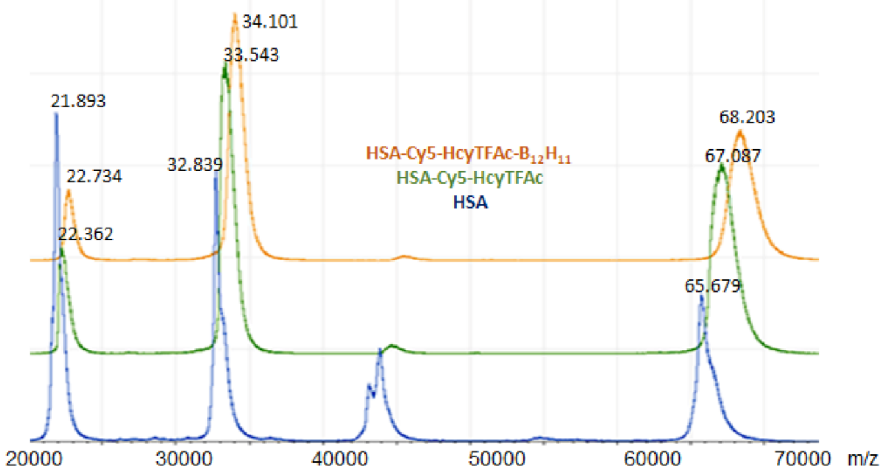

Figure S1. Characterizations of multifunctional human serum albumin conjugates. MALDI-ToF spectra of HSA (blue line), HSA-Cy5-HcyTFAc (green line) and HSA-Cy5-HcyTFAc-$B_{12}H_{11}$ (orange line).

Table S1 Identification of specific *N*-trifluorohomocysteinylation modification sites in HSA-Cy5-HcyTFAc conjugate

Site	Sequence[a]	Peptide MW calc.	MW calc., peptide MW + …[b]		*m/z* measured	missed cleavages
			+HcyTFAc	+HcyTFAc +IAA		
Lys-199	198 L***K****CASLQK 205	949.4	1,163.4	1,220.4	1,443.5 (1,402.4 + Cl^-, exchange H^+ to Li^+)	1
Lys- 414	414 ***K****VPQVSTPTLVEVSR 428	1,639.7	1,853.7	1,910.7	2,138.6 (2,092.7 + $2F^-$, exchange $2H^+$ to $2Li^+$)	1
Lys-557/560	546 AVMDDFAAFVE***K****CC***K*** *ADDK 564	2,218.9	2,432.9	2,489.9	2,519.7 (2,489.9 + Na^+, +H^+ exchange H^+ to Li^+)	2

[a]Peptide *MW* range 500-5000 Da. Trypsin does not cut after modified lysine residues, thus HcyTFAc-Lys residue cannot be present at the C-terminus (There is no peptides with the modification at the C-terminus); ***K**** lysine residues carrying *N*-linked HcyTFAc;
[b]*MW*: HcyTFAc 214 Da, IAA 57 Da. SH group of the Hcy is less reactive than cysteine SH group, that's why IAA fragment can be absent in peptide;
[c]One IAA residue or two IAA residues *MW* change, because of the presence of two SH groups in cysteine and HcyTFAc.

The molecular mass of our HSA A3782 (Sigma-Aldrich) in our mass experiments averaged 65.679 kDa. Based on the data of mass spectrometry analysis, there are three fluorinated tags conjugated to HSA. The HSA-Cy5-Hcy-TFAc conjugate had a measured molecular mass of 67.087 kDa and 33.543 kDa for the double charged protein. The difference between the homocysteinylated and native species is 1408 Da, which corresponds to ~ three *N*-homocysteinyl moieties, *N*-linked by amide linkages to ~ three Lys of HSA (*N*-Lys-Hcy-TFAc; 214 Da) plus one dye moiety on Cys34 (S-Cys-Cy5; 766.4 Da).

The *N*-Homocysteinylated residues were identified by MALDI-TOF/TOF mass spectrometry of peptide mixtures formed via trypsinolysis of modified HSA. The *N*-homocysteinylation of HSA-Cy5 by HTL-TFAc can modify three of the 59 lysine residues. According to MALDI-ToF MS data (Table S1), the Hcy-TFAc residue was attached to Lys-199, Lys-414, Lys-557/560 residues of HSA.

4.2. HSA-Cy5-HcyAc conjugate

The molecular mass of our HSA A3782 (Sigma-Aldrich) in our mass experiments averaged 65.640 kDa. The HSA-Cy5-HcyAc conjugate had a measured molecular mass of 66.692 kDa and 33.347 kDa for the double charged protein. The difference between the homocysteinylated and native species is 1052 Da. which corresponds to ~ two *N*-homocysteinyl moieties, *N*-linked by amide linkages to ~ two Lys of HSA (N-Lys-Hcy-TFAc; 214 Da) plus one dye moiety on Cys34 (S-Cys-Cy5; 766.4 Da).

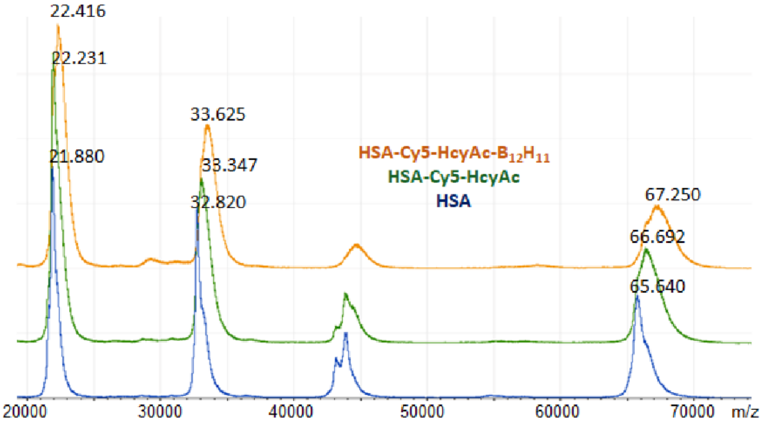

Figure S2. Characterizations of multifunctional human serum albumin conjugates. MALDI-ToF spectra of HSA (blue line), HSA-Cy5-HcyAc (green line) and HSA-Cy5-HcyAc-$B_{12}H_{11}$ (orange line).

5. Gel retardation assay of boron-albumin conjugates

Human serum albumin conjugates were analyzed by sodium dodecyl sulfate polyacrylamide gel electrophoresis using 7 % PAAG under Laemmli [8] condition without the addition of DTT or DTT with subsequent Coomassie Brilliant Blue (BioRad) staining. Quantitative data were obtained by digitizing the gel using GelPro Analyzer software (Media Cybernetics) (Table S2).

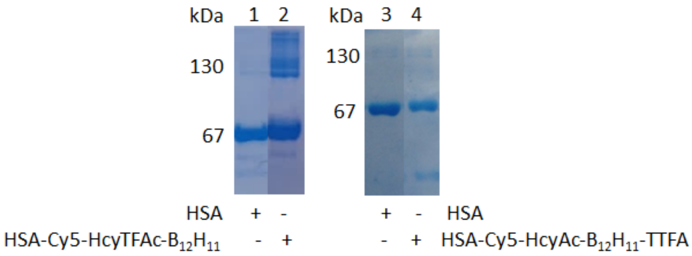

Figure S3. SDS–PAGE analysis of HSA conjugates under denaturation condition (with DTT) using 7% PAAG under Laemmli condition.

Table S2. Quantitative data of the SDS–PAGE analysis of HSA conjugates presented in Figure S3[a]

Conditions	HSA type	Monomer ~66.5 kDa	Oligomers > 130 kDa	Lower bands
Without DTT	HSA	89	7	4
	HSA-Cy5-HcyTFAc-$B_{12}H_{11}$	56.4	39.6	4
	HSA-Cy5-HcyAc-$B_{12}H_{11}$-TTFA	71	10	19
With DTT	HSA-Cy5-HcyTFAc-$B_{12}H_{11}$	100	0	0
	HSA-Cy5-HcyAc-$B_{12}H_{11}$-TTFA	95	0	5

[a]Values are given as percentage of intensity to total intensity in the lane (%). Quantitative data were obtained by digitizing the gel using GelPro Analyzer software (Media Cybernetics).

6. Circular dichroism data

Circular dichroism (CD) data were collected at 25 ºC (PBS, 7.4) with a JASCO J-600 spectrophotometer (JASCO Co., Ltd., Japan) with time constant 4 s, bandwidth 1 nm, using a 0.01 cm path length quartz cell. All CD spectra were performed in triplicate from 185 to 240 nm, and the averaged values are presented. Deconvolution of CD spectra was carried out according to Louis-Jeune, et al. [9] with α-helix and β-sheet percent errors being about 3%.

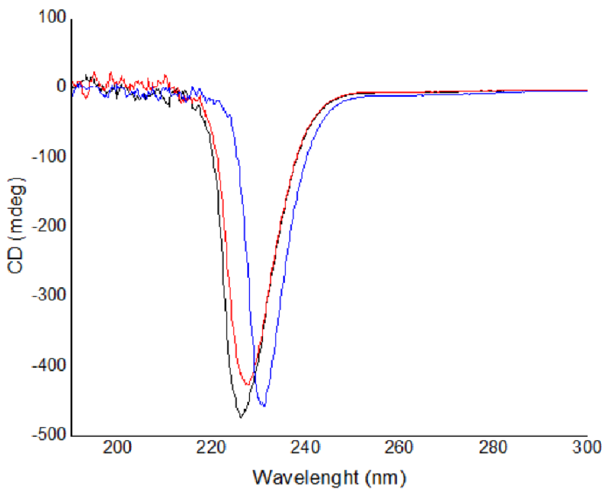

Figure S4. Circular dichroism (CD) spectra of the unmodified HSA (blue), HSA-Cy5-Hcy-TFAc (red), HSA-Cy5-Hcy-TFAc-$B_{12}H_{11}$ (black).

Table S3. Secondary structures calculated by deconvolution of the CD spectra shown in Figure S4

HSA type	α-Helix (%)	β-Sheet (%)
HSA	65.02	10.15
HSA-Cy5-HcyTFAc	65.02	10.15
HSA-Cy5-HcyTFAc-$B_{12}H_{11}$	64.93	10.49

7. NMR data.

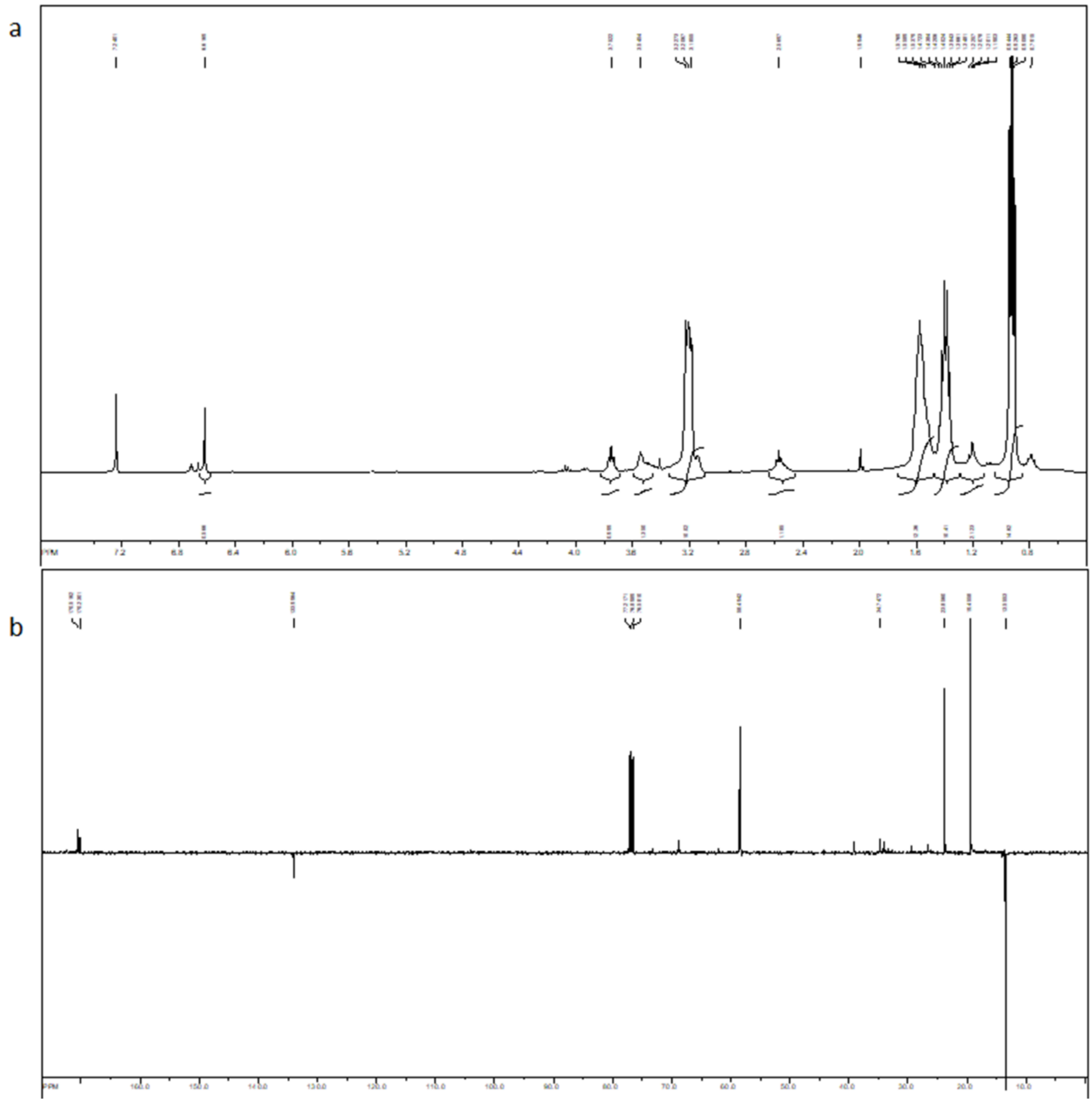

Figure S5. NMR spectra of maleimide-conjugating closo-dodecaborate tetramethylammonium form ($B_{12}H_{11}$-mal (Bu_4N^+)) in $CDCl_3$: a) ^{1}H NMR, the chemical shifts are referred to the resonance of $CDCl_3$ at 7.25 ppm; b) ^{13}C NMR, the chemical shifts are referred to the resonance of TMS.

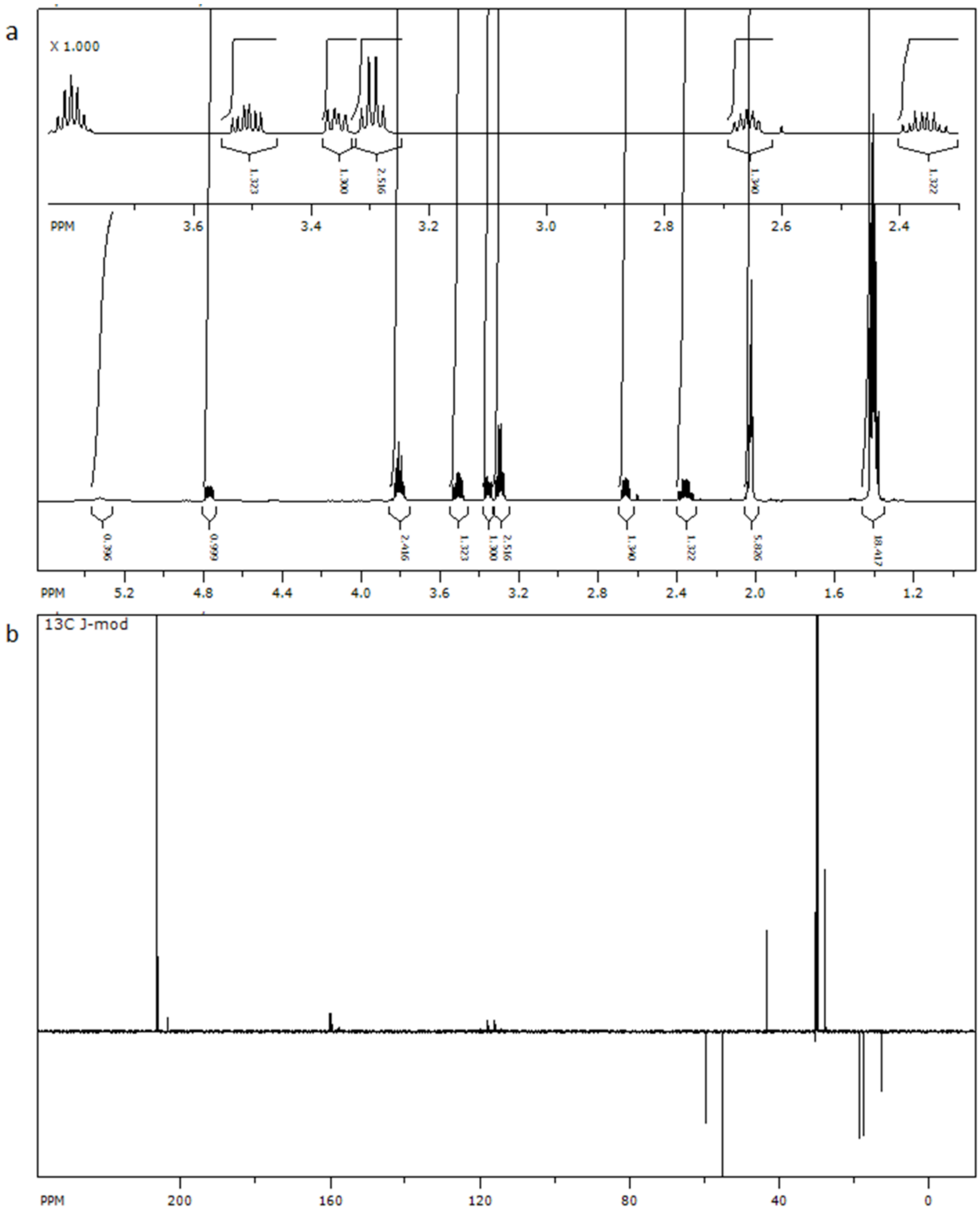

Figure S6. NMR spectra of 2,2,2-trifluoro-N-(2-oxotetrahydrothiophen-3-yl)acetamide (HTLTFAc) in acetone-D6: a) ^{1}H NMR, the chemical shifts are referred to the resonance of acetone-D6 at 2.07 ppm; b) ^{13}C NMR, the chemical shifts are referred to the resonance of TMS.

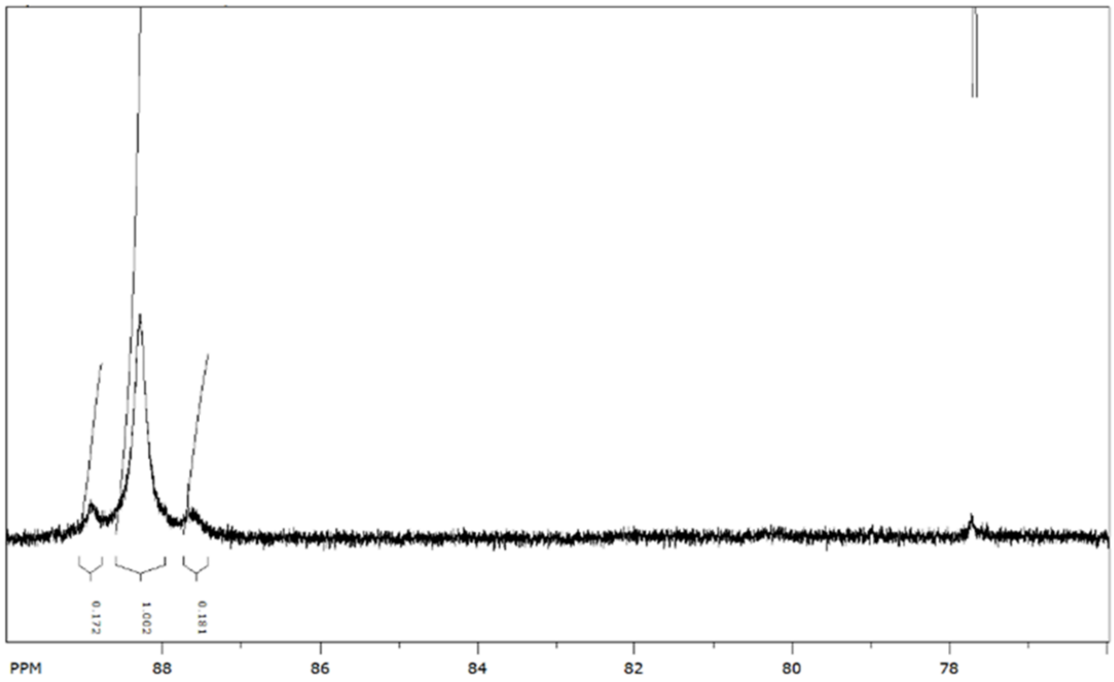

Figure S7. ^{19}F NMR spectrum (at 282.4 MHz) of HSA-Cy5-HcyAc-$B_{12}H_{11}$-TTFA (0.6 mM) in PBS buffer (pH 7.4, to provide deuterium lock, D_2O was added to 20% of the total volume) at 37°C. The chemical shifts are referred to the resonance of C_6F_6 at 0.0 ppm.

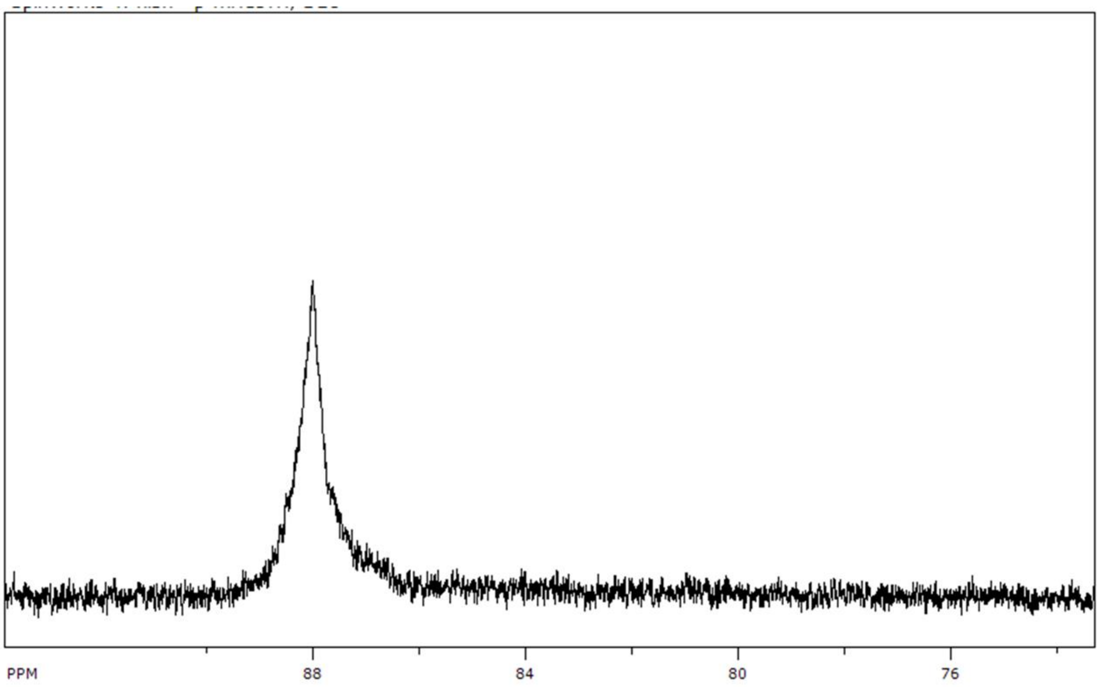

Figure S8. ^{19}F NMR spectrum (at 282.4 MHz) of HSA-Cy5-HcyTFAc-$B_{12}H_{11}$ (0.3 mM) in PBS buffer (pH 7.4, to provide deuterium lock, D_2O was added to 20% of the total volume) at 37°C. The chemical shifts are referred to the resonance of C_6F_6 at 0.0 ppm.

REFERENCES

1. Janatova, J.; Fuller, J.K.; Hunter, M.J. The heterogeneity of bovine albumin with respect to sulfhydryl and dimer content. *J. Biol. Chem.* **1968**, *243*, 3612–3622.

2. Peters, T. Jr. The Albumin molecule: its structure and chemical properties. In *All about Albumin: Biochemistry, Genetics, and Molecular Applications.* Academic Press, San Diego, USA, 1996; p. 432.
3. Kikuchi, S.; Kanoh, D.; Sato, S.; Sakurai, Y.; Suzuki, M.; Nakamura, H. Maleimide-functionalized *closo*-dodecaborate albumin conjugates (MID-AC): Unique ligation at cysteine and lysine residues enables efficient boron delivery to tumor for neutron capture therapy. *J. Control. Release.* **2016**, *237*, 160–167.
4. Semioshkin, A.; Nizhnik, E.; Godovikov, I.; Starikova, Z.; Bregadze, V. Reactions of oxonium derivatives of $[B_{12}H_{12}]^{2-}$with amines: Synthesis and structure of novel B_{12}-based ammonium salts and amino acids. *J. Organomet. Chem.* **2007**, *692*, 4020–4028.
5. Sivaev, I.B.; Semioshkin, A.A.; Brellochs, B.; Sjoberg, S.; Bregadze V.I. Synthesis of oxonium derivatives of the dodecahydro-closo-dodecaborate anion $[B_{12}H_{12}]^{2-}$. Tetramethylene oxonium derivative of $[B_{12}H_{12}]^{2-}$ as a convenient precursor for the synthesis of functional compounds for boron neutron capture therapy. *Polyhedron.* **2000,** *19*, 627–632.
6. Chubarov, A.V.; Shakirov, M.M.; Koptyug, I.V.; Sagdeev, R.Z.; Knorre, D.G.; Godovikova, T.S. Synthesis and characterization of fluorinated homocysteine derivatives as potential molecular probes for 19F magnetic resonance spectroscopy and imaging. *Bioorg. Med. Chem. Lett*. **2011**, *21*, 4050-4053.
7. Chubarov, A.S.; Zakharova, O.D.; Koval, O.A.; Romaschenko, A.V.; Akulov, A.E.; Zavjalov, E.L.; Razumov, I.A.; Koptyug, I.V.; Knorre, D.G.; Godovikova, T.S. Design of protein homocystamides with enhanced tumor uptake properties for ^{19}F magnetic resonance imaging. *Bioorg. Med. Chem.* **2015,** *23*, 6943–6954.
8. Cleveland, D. W.; Fischer, S. C.; Kirschner, M. W.; Laemmli, U. K. Peptide mapping by limited proteolysis in sodium dodecyl sulfate and analysis by gel electrophoresis. *J. Biol. Chem.* **1977**, *252*, 1102–1106.
9. Louis-Jeune, C.; Andrade-Navarro, M.A.; Perez-Iratxeta, C. Prediction of protein secondary structure from circular dichroism using theoretically derived spectra. *Proteins.* **2012**, *80*, 374 – 381.
